# Therapeutic Potential of Liraglutide for Diabetes–Periodontitis Comorbidity: Killing Two Birds with One Stone

**DOI:** 10.1155/2022/8260111

**Published:** 2022-07-06

**Authors:** Man Yang, Yunqing Pang, Minyu Pei, Yuanyuan Li, Xuemin Yuan, Rongbing Tang, Jing Wang

**Affiliations:** ^1^School/Hospital of Stomatology, Lanzhou University, Lanzhou, China; ^2^Key Laboratory of Stomatology of the State Ethnic Affairs Commission, China; ^3^Hospital of Stomatology, Xi'an Jiaotong University, Xi'an, China; ^4^Gansu Province Clinical Research Center for Oral Diseases, Gansu Province, China

## Abstract

**Background:**

The relationship between diabetes and periodontitis is bidirectional, and there is now consensus that periodontitis and diabetes are comorbid. There is a quest for a drug that can be used to treat both conditions simultaneously. This study evaluated the anti-inflammatory and osteoprotective effects of liraglutide (LIRA) on periodontitis in diabetic rats.

**Methods:**

Male Wistar rats (*n* = 46) were randomly divided into four groups: control group (*n* = 8), LIRA group (*n* = 8), diabetes-associated periodontitis+0.9% saline group (diabetic periodontitis (DP)+NaCl group, *n* = 15), and diabetes-associated periodontitis+LIRA group (DP+LIRA group, *n* = 15). LIRA treatment lasted for 4 weeks (300 *μ*g/kg/d) after establishment of a rat model of DP. The expression of IL-6, TNF-*α*, and IL-1*β* was detected by enzyme-linked immunosorbent assay. The morphological changes of periodontal tissues were observed by hematoxylin–eosin staining. The absorption of alveolar bone and its ultrastructural changes were observed by histomorphometry and microcomputed tomography. The expression of receptor activator of NF-*κ*B ligand (RANKL) and osteoprotegerin (OPG) in alveolar bone was detected by immunohistochemistry. The levels of Runx2 mRNA and ALP mRNA in the gingival epithelium were examined by quantitative real-time polymerase chain reaction.

**Results:**

LIRA decreased alveolar bone resorption, improved the microstructure of alveolar bone, and reduced periodontal inflammation and damage (*P* < 0.05). LIRA also reduced blood glucose level and inhibited the secretion of serum IL-6, TNF-*α*, and IL-1*β* (*P* < 0.05). In addition, after treatment with LIRA, the ratio of RANKL/OPG was reduced, and the expression levels of ALP mRNA and Runx2 mRNA were upregulated (*P* < 0.05).

**Conclusions:**

LIRA not only controls blood glucose level but also reduces inflammation and bone loss and enhances osteogenic differentiation in diabetes-associated periodontitis. Those indicate that LIRA may be used as a potential medicine for the adjunctive therapy of diabetes-periodontitis comorbidity.

## 1. Introduction

Periodontitis is a chronic inflammatory disease caused by periodontal infection and local factors. It is the main cause of tooth loss [1]. Periodontitis is closely associated with a variety of systemic diseases, such as cardiovascular disease, osteoporosis, and especially diabetes [2, 3]. There is a bidirectional relationship between periodontitis and diabetes. A previous meta-analysis has demonstrated that the incidence of periodontitis in patients with diabetes is three times higher than that in individuals without diabetes [4]. Meanwhile, the severity of periodontitis can affect blood glucose control in patients with diabetes, and patients with moderate or severe periodontitis are at a significantly increased risk of developing diabetes in the future [5]. There is now a consensus that periodontal disease and diabetes are comorbid [6]. Therefore, it is necessary to find a drug for the treatment of diabetes–periodontitis comorbidity, so as to simultaneously treat both conditions.

There has been much research interest in antidiabetes medicines, whose anti-inflammatory properties have the potential to improve periodontitis. Bak et al. [7] found that metformin decreased inflammatory infiltration of periodontium and alveolar bone loss in rats with ligature-induced periodontitis. Pradeep et al. [8] applied metformin gel to patients with periodontitis and confirmed the therapeutic effect of metformin on periodontitis. Guo et al. [9] analyzed the effect of exdendin-4 on human periodontal ligament stem cells (PDLSCs) under high-glucose environment and showed that exdendin-4 alleviated the inhibition of high glucose on the osteogenic differentiation of PDLSCs and directly promoted the osteogenic differentiation of normal PDLSCs. The results showed that exdendin-4 may be applied in the treatment of diabetic periodontitis (DP). Nishikawa et al. [10] established streptozotocin- (STZ-) induced experimental periodontitis in diabetic rats and investigated the effect of insulin on DP. The results showed that insulin could improve inflammatory cell infiltration and expression of inflammatory cytokine genes and reduced the loss of alveolar bone. In addition, sulfonylurea drugs alleviate periodontitis possibly via direct mitigation of inflammation or indirectly via an antidiabetic effect [11]. Our previous experiments have also shown that hypoglycemic drug liraglutide (LIRA) can promote osteogenesis and inhibit the inflammatory reaction of human periodontal ligament cells (hPDLCs) stimulated by lipopolysaccharide, and confirm the therapeutic effect of LIRA on periodontitis in rats [12, 13]. However, more studies are needed to verify whether LIRA has the expected therapeutic effect on DP.

In this study, type 2 diabetes in rats was induced by high-fat diet combined with low-dose injection of STZ, and then, periodontitis was induced by ligation. Based on the above methods, we established an animal model of diabetes-related periodontitis to investigate the therapeutic effect of systemic LIRA on diabetes–periodontitis comorbidity.

## 2. Methods

### 2.1. Establishment of an Experimental Model of Diabetes-Associated Periodontitis

Forty-six male Wistar rats (aged 5 weeks) were randomly divided into four groups: control group (*n* = 8), LIRA group (*n* = 8), diabetes-associated periodontitis+0.9% saline group (DP+NaCl group, *n* = 15), and diabetes-associated periodontitis+LIRA group (DP+LIRA group, *n* = 15).

Diabetes was induced in accordance with the method described in the literature [14]. To induce diabetes, Wistar rats ate a high-fat diet (65% basic diet, 10% lard, 20% sucrose, 2.5% cholesterol, and 1% sodium cholate) and were injected with low-dose STZ (35 mg/kg, Sigma-Aldrich, USA). The rats were identified as diabetic ones, whose fasting blood glucose (FBG) level was higher than 11.1 mmol/L for three consecutive days. Periodontitis was induced at the same time after the successful establishment of the diabetes model. Orthodontic ligation wires were ligated in the neck of the rat's upper left first molar, and medical sutures were added to induce periodontitis. The ligatures were removed after 4 weeks, and LIRA (300 *μ*g/kg/d; Novo Nordisk, Denmark) was injected intraperitoneally for 4 weeks.

Animal care and experiments were carried out according to the protocol approved by the Ethical Research Committee of Lanzhou University School of Stomatology. This work was also conducted in accordance with the guidelines approved by the Council of the American Psychological Society (2010) for the use of experimental animals.

### 2.2. FBG and Body Weight Determination

After fasting for 10 hours, blood was sampled from the caudal vein. FBG levels were measured by a Roche Glucometer (Roche, Germany). Throughout the study, body weights and FBG levels were detected to ensure the maintenance of diabetes.

### 2.3. Oral Glucose Tolerance Test (OGTT)

Oral glucose (2 g/kg body weight) was administered by tube feeding after fasting for 12 hours. After glucose application, blood glucose levels were detected with Accu-Chek active system (Roche, Germany) for 0, 60, 90, 120, and 180 minutes.

### 2.4. Histological Evaluation

Calcium in the gingival tissue and the left maxillary bone was removed in 10% ethylenediaminetetraacetic acid for ten weeks and embedded in paraffin. The specimens were sectioned (4 *μ*m thick) across the mesiodistal direction of the first molar and were observed with hematoxylin–eosin (HE) staining. We observed inflammatory infiltration of gingival tissues and the destruction of alveolar bone under an optical microscope. At the same time, the left maxillary alveolar bone was stained with methylene blue to observe the resorption of alveolar bone.

The slides were deparaffinized, rehydrated, and incubated in 3% hydrogen peroxide for 10 minutes to reduce endogenous peroxidase activity. Antigen retrieval was accomplished by incubation in citrate buffer at high temperature and high pressure. Then, the slides were immersed in a serum blocking reagent at 37°C for 15 minutes to block nonspecific binding sites. Next, the samples were incubated overnight with anti-osteoprotegerin antibody (OPG, bs-0431R, Bioss, China) (1 : 300) and anti-receptor activator of NF-*κ*B ligand antibody (RANKL, YM3065, ImmunoWay, USA) (1 : 100) in a humidification chamber at 4°C. Then, the slides were incubated with goat anti-rabbit immunoglobulin G (IgG) secondary antibody (SP-9001, ZSGB-BIO, China) labeled with biotin at 37°C for 30 minutes and washed with the antibiotic-biotin-peroxidase complex. Finally, 3,3-diaminobenzidine tetrachloride hydrate (DAB) (ZSGB-BIO, China) was used to develop stain and hematoxylin was used to counterstain. The procedure for negative controls was the same, except for the use of phosphate buffer instead of a primary antibody. A digital camera (Canon, Canon Ltd., Japan) was used to capture images under a microscope. The number of specific positively stained cells (brown-yellow granules) was calculated in periodontal tissue, including alveolar bone and gingival tissue. For each sample, 12 fields of view were randomly selected, and the average number of positively stained cells per square millimeter was determined.

### 2.5. Microcomputed Tomography (Micro-CT) Evaluation

Wistar rats were euthanized, and left maxillary specimens were dissected and fixed with 4% paraformaldehyde for 24 hours and then stored in 70% alcohol for micro-CT (Quantum GX, PerkinElmer, USA) scanning. The extent of alveolar bone resorption was quantified by three-dimensional (3D) reconstruction. Furthermore, the images of alveolar bone around the left maxillary first molar were selected as the region of interest (ROI), and the following microarchitectural parameters were analyzed: bone mineral density (BMD), trabecular number (Tb.N), trabecular bone volume fraction (Tb.BV/TV), trabecular thickness (Tb.Th), cortical thickness (Ct.Th), and trabecular separation (Tb.Sp).

### 2.6. Enzyme-Linked Immunosorbent Assay (ELISA)

Whole blood samples were collected by cardiac puncture, kept for 2 hours at room temperature, and centrifuged at 1000 rpm for 15 minutes; the supernatant was obtained for detection. Levels of IL-6, TNF-*α*, and IL-1*β* proteins were detected by ELISA kits (Multi Sciences, China). Data were presented in picogram/milliliter, and all measurements were made in triplicate and averaged.

### 2.7. Quantitative Real-Time Polymerase Chain Reaction (qRT-PCR)

Total RNA was extracted from the gingival tissue by using TRIzol reagent (Invitrogen, Carlsbad, CA, USA) in accordance with the manufacturer's instructions, and mRNA was reverse-transcribed by using the RT reagent kit (Takara, Japan). Then, qRT-PCR was performed using SSoAdvanced SYBR Green Supermix. Primer sequences used for qRT-PCR were as follows: Runx2, forward 5′-CATGGCCGGGAATGATGAG-3′ and reverse 5′-TGTGAAGACCGTTATGGTCAAAGTG-3′; ALP, forward 5′-CATCGCCTATCAGCTAATGCACA-3′ and reverse 5′-ATGAGGTCCAGGCCATCCAG-3′; and *β*-actin, forward 5′-GGAGATTACTGCCCTGGCTCCTA-3′ and reverse 5′-GACTCATCGTACTCCTGCTTGCTG-3′. The comparative expression of Runx2 and ALP was computed via the 2^−*ΔΔ*Ct^ approach with *β*-actin as the internal control.

### 2.8. Statistical Analysis

All statistical analyses were performed by using a statistical analysis program (SPSS, Chicago, IL, USA). Data are represented as the mean ± standard deviation (SD). Statistical comparisons were made by one-way ANOVA, and *P* values < 0.05 were considered statistically significant.

## 3. Results

### 3.1. Effects of LIRA on Body Weight and Blood Glucose Level

**Figure 1:**
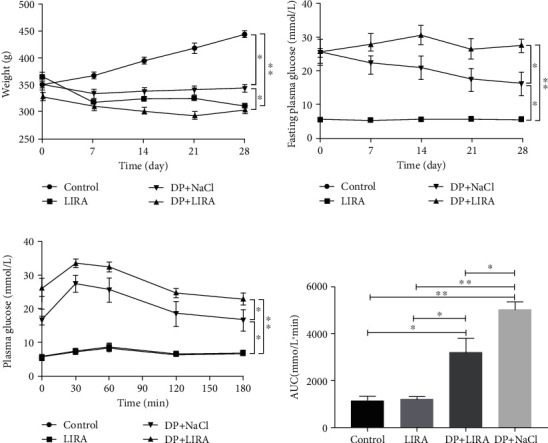
Effects of LIRA on body weight and blood glucose levels in rats. (a) Changes in the body weight during LIRA administration for 4 weeks. (b) FBG levels of rats during LIRA administration for 4 weeks. (c) OGTT was performed after LIRA treatment. (d) The area under the curve (AUC) of rats after LIRA treatment. ^∗^*P* < 0.05; ^∗∗^*P* < 0.01.

The weight of DP rats was lower than that of the control group. After LIRA treatment for 4 weeks, the weight of DP rats increased (*P* < 0.05), but it was still lower than that in the control group (Figure 1(a)). The results of FBG determination and OGTT showed that the blood glucose levels of DP rats decreased after LIRA treatment, but the levels were still higher than those in the control group. The dose of LIRA had no effect on the blood glucose levels of normal rats (Figures 1(b)–1(d)).

### 3.2. Anti-Inflammatory Effect of LIRA on DP

**Figure 2:**
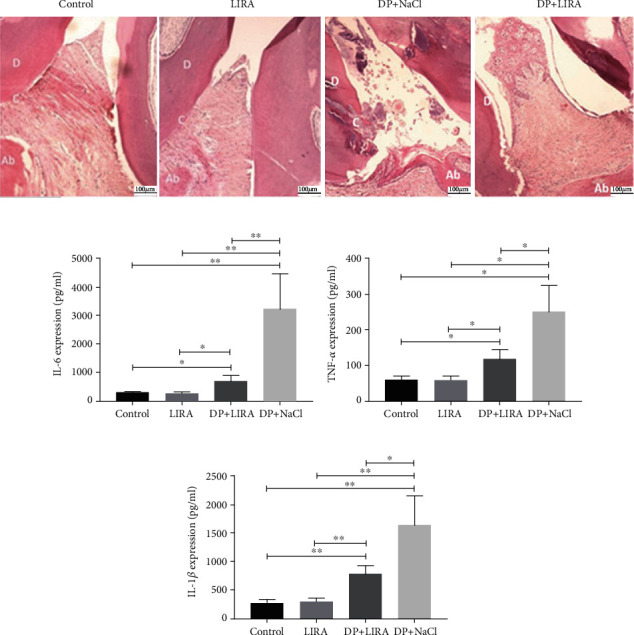
Anti-inflammatory effect of LIRA on DP. (a) HE staining images of periodontal tissues. Scale bar, 100 *μ*m. D: dentin, C: cementum, Ab: alveolar bone. (b) The expression of IL-6 in the serum of rats. (c) The expression of TNF-*α* in the serum of rats. (d) The expression of IL-1*β* in the serum of rats. ^∗^*P* < 0.05; ^∗∗^*P* < 0.01.

We observed HE-stained sections between the maxillary first and second molars under a light microscope. The gingival epithelium in the control and LIRA groups was intact, without abnormal changes in connective tissue and loss of attachment. In contrast, in the DP+LIRA group and the DP+NaCl group, we observed a high number of inflammatory cells in the sulcus epithelium, the partially degenerated collagen fibers, and the junctional proliferating epithelium toward the root. The inflammatory status of the DP+LIRA group was significantly better than that of the DP+NaCl group (Figure 2(a)). The blood levels of the inflammatory factors were detected by ELISA. The results showed that the levels of TNF-*α*, IL-6, and IL-1*β* in DP were significantly increased compared with the control group and then dramatically decreased after LIRA treatment (Figures 2(b)–2(d)). These data suggest that LIRA can inhibit the inflammatory response of DP.

### 3.3. Bone Protective Effect of LIRA on DP

**Figure 3:**
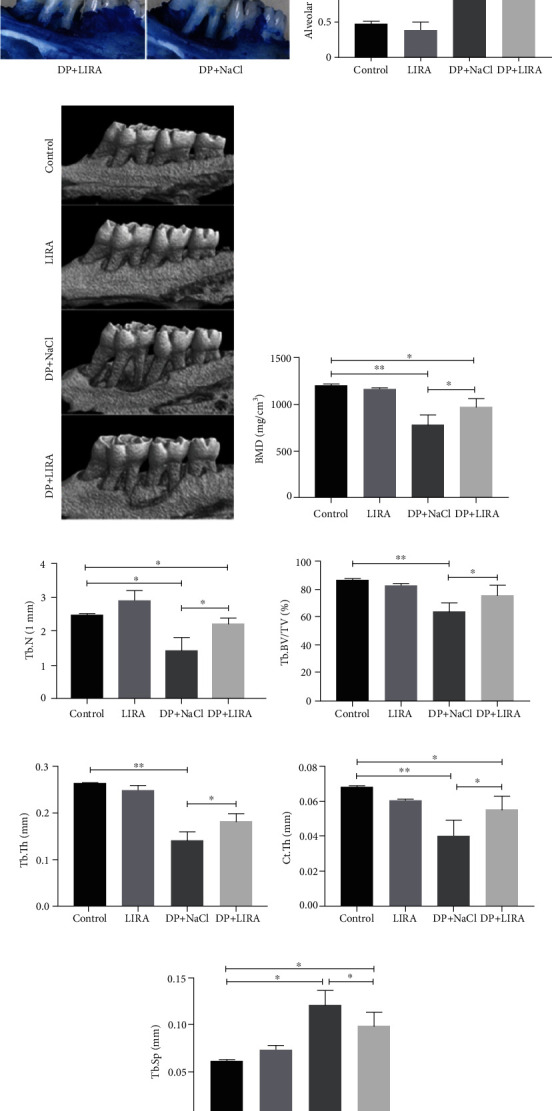
Effects of LIRA on the alveolar bone. (a) Methylene blue–stained left maxillary bone. (b) Quantitative analysis of alveolar bone resorption. (c) Representative micro-CT images by 3D reconstruction. (d) Changes in the bone mineral density (BMD). (e) Changes in trabecular bone number (Tb.N). (f) Changes in trabecular bone volume fraction (Tb.BV/TV). (g) Changes in trabecular thickness (Tb.Th). (h) Changes in cortical thickness (Ct.Th). (i) Changes in trabecular separation (Tb.Sp). ^∗^*P* < 0.05; ^∗∗^*P* < 0.01.

First, we analyzed the effects of LIRA on alveolar bone resorption of the maxillary first molar. The results of rat maxilla stained with hyacinth blue suggested that alveolar bone resorption was more obvious in DP, while it improved after LIRA treatment (Figures 3(a) and 3(b)). Furthermore, we used micro-CT to analyze the alveolar bone by converting the selected ROI into a 3D target volume. The results of 3D analysis of maxillary bone morphological parameters were similar to the results of methylene blue staining (Figures 3(c)–3(i)). These data demonstrate that LIRA not only inhibits the reduction of alveolar bone height in DP but also improves bone quality and bone density of the alveolar bone.

**Figure 4:**
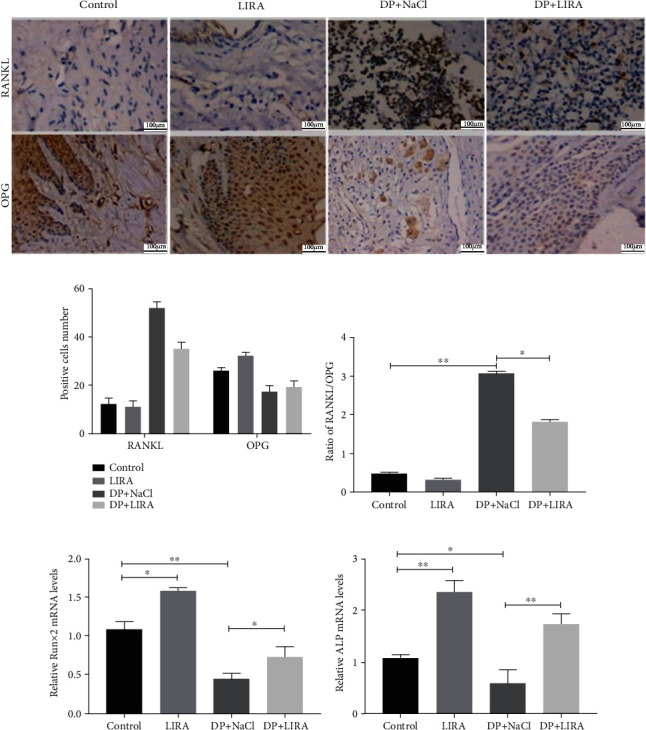
Effects of LIRA on bone regeneration by regulating RANKL/OPG. (a) Immunohistochemical staining of RANKL and OPG in periodontal tissues. Scale bar, 100 *μ*m. (b, c) Quantitative analysis of RANKL/OPG ratios. (d) The expression of Runx2 mRNA. (e) The expression of ALP mRNA. ^∗^*P* < 0.05; ^∗∗^*P* < 0.01.

Second, we assessed the effect of LIRA on the expression of RANKL and OPG in periodontal tissues by immunohistochemistry. The intensity of immunostaining of RANKL in periodontal tissue was in the following order: DP + NaCl group > DP + LIRA group > control group and LIRA group. The intensity of immunostaining of OPG was in the following order: control group and LIRA group > DP + LIRA group > DP + NaCl group. LIRA attenuated the immunostaining intensity of RANKL and increased the immunostaining intensity of OPG in the alveolar bone of DP rats (Figures 4(a) and 4(b)). Meanwhile, quantitative analysis of the RANKL/OPG ratio showed that the RANKL/OPG ratio of the DP+LIRA group was significantly lower than that of the DP+NaCl group (*P* < 0.05) (Figure 4(c)), indicating that LIRA can reduce the osteoclast activity of alveolar bone.

Finally, the expression of Runx2 mRNA and ALP mRNA in the gingival tissue of DP rats was detected by qRT-PCR (Figures 4(d) and 4(e)). We found that the expression levels of Runx2 mRNA and ALP mRNA in the DP+NaCl group were significantly lower than those in the control group (*P* < 0.05). In contrast, after LIRA treatment, the expression levels of Runx2 mRNA and ALP mRNA were upregulated (*P* < 0.05). More interestingly, the expression levels of Runx2 mRNA and ALP mRNA in the LIRA group were also higher than those in the control group (*P* < 0.05). These results suggest that LIRA not only promotes the osteogenic differentiation of periodontal tissue in DP rats but also increases the ability of healthy rats to resist the risk of alveolar bone resorption.

## 4. Discussion

The bidirectional association between periodontitis and diabetes has been well researched. It is generally believed that diabetes affects periodontitis in four ways, including hyperinflammatory response to infection, oxidative stress, bone metabolism disorders, and accumulation of advanced glycation end products (AGEs) and their receptors (RAGE) [15]. Diabetes can promote the systemic inflammatory response, increase the expression of inflammatory factors in the blood, promote the destruction of periodontal tissue, and hinder the repair of periodontal tissue [3, 16]. The elevation of blood glucose levels in patients with diabetes can cause a large number of free radicals to accumulate rapidly, leading to the disturbance of local microcirculation and increasing the susceptibility to periodontal tissue inflammation [17]. Moreover, the host immunity in diabetes decreases, and microvascular lesions can reduce the local tissue oxygen concentration [18], cause the imbalance of oral flora [19], accelerate the resorption of alveolar bone, and aggravate the destruction of periodontal tissue. In addition, overexpression of AGEs/RAGE exacerbates the inflammatory response, thereby aggravating the destruction of periodontal tissue in patients with diabetes [20]. Periodontitis can aggravate the systemic inflammatory response through persistent bacteremia and elevate the levels of proinflammatory cytokines such as IL-6 and TNF-*α* [21], reduce the sensitivity of target cells to insulin, promote insulin resistance [22], and interfere with the normal metabolism of the body, thereby aggravating the metabolic disorder of diabetes. In brief, periodontitis combined with diabetes aggravates the pathology of these two diseases, so it is necessary to explore a drug that can treat these two diseases simultaneously.

In this study, we investigated the therapeutic effect of LIRA on experimental periodontitis in diabetic rats and established the DP model induced by long-term high-fat diet and low-dose STZ injection combined with ligation. Increased FBG level, abnormal glucose tolerance, periodontal inflammatory cell infiltration, and alveolar bone loss confirmed the successful establishment of the DP model. This method well mimics the disease process of clinical diabetes-associated periodontitis. High-fat diet induces obesity in animals, and low-dose injection of STZ can induce apoptosis of some islet *β*-cells, resulting in relatively insufficient insulin secretion and abnormal glucose metabolism, thereby simulating the clinical pathogenesis of type 2 diabetes [23]. Ligation-induced periodontitis is also the most classical method. In this study, the orthodontic ligation wires were ligated in the neck of the rat's upper left first molar, and medical sutures were added to the model, which is difficult to slip and convenient for long-term observation [24, 25].

Inflammation is a common characteristic of periodontitis and diabetes. Therefore, after the successful establishment of the DP model, our study first analyzed the effect of LIRA on the inflammatory response of DP rats. Results of HE showed that LIRA could decrease inflammatory cell infiltration in periodontium. Meanwhile, LIRA significantly reduced the levels of proinflammatory cytokine such as IL-6, TNF-*α*, and IL-1*β* in serum of DP rats. In short, LIRA can inhibit the inflammatory response of DP. LIRA is a glucagon-like peptide-1 (GLP-1) analogue that mainly binds with GLP-1 receptor (GLP-1R) to exert its effects, such as lowering blood glucose levels, inhibiting inflammation, and promoting bone formation. Our previous study found that the expression of GLP-1R was detected on hPDLCs, and LIRA could inhibit the expression of IL-6 and TNF-*α* on the experimental periodontitis [12, 13]. Therefore, the inhibitory effect of LIRA on periodontal inflammation in DP rats is derived from the indirect hypoglycemic effect of LIRA and the direct inhibitory effect of LIRA on periodontal proinflammatory factors.

The treatment of diabetes-associated periodontitis still depends on the regeneration of alveolar bone. RANKL-RANK-OPG participates in the regulation of bone homeostasis, and RANKL interacts with its receptor RANK to induce osteoclast formation and activity, while OPG competes with RANK to attenuate this effect [26]. The RANKL/OPG ratio of patients with DP is adversely affected by poor blood glucose control, leading to increased osteoclast production [27]. In this study, high levels of inflammatory cell infiltration, increased number of osteoclasts, decreased number of osteoblasts, and an increased RANKL/OPG ratio were observed in the periodontal tissues of DP rats. These findings are consistent with previous studies [27, 28] and indicate that diabetes can aggravate the alveolar bone resorption in periodontitis by increasing the level of inflammation, increasing osteoclast proliferation, and reducing osteoblast formation. After LIRA treatment, the ratio of RANKL/OPG significantly decreased; the absorption of alveolar bone was significantly reduced; and the bone microstructure of alveolar bone was significantly improved. To sum up, we demonstrated that LIRA can reduce osteoclast activity by regulating RANKL/OPG, thereby reducing alveolar bone resorption.

Furthermore, LIRA can promote the expression of Runx2 mRNA and ALP mRNA in DP gingival tissue. ALP is an exoenzyme secreted by osteoblasts, which plays an important role in bone formation and mineralization. ALP is a marker of early osteogenesis [29]. Runx2 is an osteogenic transcription factor that regulates transcription of multiple factors and extracellular matrix protein secretion, which can promote osteogenic differentiation and inhibit osteoclast formation. Runx2 plays a vital role in osteogenic differentiation and bone formation [30]. The increased expression of Runx2 mRNA and ALP mRNA confirmed the osteogenic effect of LIRA on periodontal tissue in DP rats. More interestingly, LIRA also increased the expression of Runx2 mRNA and ALP mRNA in normal rats, suggesting that LIRA may have the potential to prevent alveolar bone resorption. These findings are consistent with those from previous studies [31], where LIRA increased lumbar spine trabecular bone volume, number, and thickness in ovariectomized mice, suggesting a protective effect of LIRA on nondiabetic bone loss, which is not due to glucose regulation.

## 5. Conclusion

In conclusion, we successfully established a model of diabetes-related periodontitis in rats. Intraperitoneal administration of LIRA not only reduces blood glucose level but also reduces the inflammatory infiltration of periodontal tissue caused by periodontitis in diabetic rats, reduces alveolar bone loss, and improves bone tissue formation. Therefore, LIRA may be used as a potential drug for the treatment of diabetes–periodontitis comorbidity.

## Data Availability

The full data used to support the findings of this study are available from the corresponding author upon request.
